# Latanoprost PF vs. Bimatoprost PF: Which Treats the Ocular Surface Better?

**DOI:** 10.3390/jcm12216732

**Published:** 2023-10-25

**Authors:** Georgios S. Dimtsas, Anastasia Tsiogka, Marilita M. Moschos

**Affiliations:** First Department of Ophthalmology, National and Kapodistrian University of Athens, “G. Gennimatas” General Hospital, 11527 Athens, Greece

**Keywords:** Latanoprost, Bimaroptost, MMP-9, glaucoma

## Abstract

(1) Background: The current study aimed to compare two of the most frequently prescribed preservative-free (PF) antiglaucoma drops, (Latanoprost PF vs. Bimatoprost PF) in promoting OSD in patients with POAG. (2) Methods: In this prospective study, 44 eyes from 44 participants were included. In the control group we enrolled 24 eyes, 11 eyes treated only with Latanoprost PF were enrolled in the Latanoprost PF group, and 9 eyes treated only with Bimatoprost PF in the Bimatoprost PF group. In all eyes, we evaluated the ocular levels of MMP-9 using the InflammaDry kit. We also performed Schirmer’s test and the TBUT test. (3) Results: We found elevated ocular levels of MMP-9 (>40 ng/mL) in the Bimatoprost PF group (88.89% of the participants) compared to the control (8.33%) and the Latanoprost PF group (27.27%), and the difference was statistically significant (*p* < 0.001). The Schirmer’s test values were statistically significantly lower in the Bimatoprost PF group compared to the other two groups. Additionally, the TBUT values were lower in the Bimatoprost PF group compared to the control group, and the difference was statistically significant. (4) Conclusions: Latanoprost PF eye drops treat the ocular surface better and they do not induce overexpression of MMP-9, a molecule that is related to OSD.

## 1. Introduction

Glaucoma is defined as a group of neuropathies that result in progressive optic nerve atrophy, with characteristic changes in the optic head and progressive visual field defects. It is the leading cause of global irreversible blindness, and the number of worldwide glaucoma patients aged over 40 years is estimated to increase to 111.8 million in 2040 [1]. The main strategy for treating glaucoma is to control the intraocular pressure (IOP) by administering antiglaucoma eye drops for a prolonged and frequently lifelong period. Prostaglandin analogues are considered as an initial medical therapy for lowering IOP in patients with glaucoma, as they are highly efficacious, well tolerated, administered once daily, and are also relatively safe [2]. Prostaglandin analogues reduce IOP by increasing the outflow of aqueous humor [3]. Two of the most frequently prescribed prostaglandin analogues are Latanoprost 0.005% (Monoprost, THEA, Clermont-Ferrand, France) and Bimatoprost 0.03% (Lumigan, Allergan, North Chicago, IL, USA), which can be administered either in a preserved or in a preservative-free (PF) solution. Previous studies have shown that preserved formulations with Benzalkonium chloride (BAK) induce stronger ocular surface disease (OSD) symptoms compared to PF formulations [4].

We have to mention that a considerable number of patients with glaucoma experience OSD symptoms which can affect the quality of life and the adherence to therapy [5,6,7]. Evaluating the ocular surface in patients with glaucoma is an important procedure for maintaining a good quality of life and visual function. We have several tests at our disposal that can be performed in order to evaluate the condition of the tear film, such as Schirmer’s test and the Tear Break-up Time (TBUT) test. Unfortunately, those two tests do not correlate often with the patient’s clinical symptoms. The Schirmer’s test especially is unrepeatable due to the reflex tear produced by its irritating nature [8,9] and the TBUT test can be unreliable because of the use of topical anaesthetic agents which destabilize the tear film and lead to artificially accelerated TBUT values [9,10]. A novel approach to evaluating ocular surface disease was recently reported by Chotikavanich et al. [11]. They have shown that the tear-film levels of the Matrix Metalloproteinase-9 (MMP-9) molecule are elevated in patients with severe OSD, Meibomian gland dysfunction, Sjogren’s syndrome, and glaucoma [11].

MMPs are proteolytic enzymes which are produced by stressed ocular surface and glandular epithelial cells, as well as by the inflammatory cells that infiltrate those tissues [11]. MMPs play a vital role in wound healing and inflammation [12,13]. Among MMPs, MMP-9 has been found to be of central importance in cleaving epithelial basement membrane components and tight junction proteins that maintain corneal epithelial barrier function [14,15,16]. MMP-9 belongs to the gelatinase group of metalloproteinases that degrade denatured collagen; native collagens type IV, V, and VII; and elastin. The normal MMP-9 level in human tears is low (3–40 ng/mL) [17]. Elevated MMP-9 levels have been found in the tears of patients with OSD [11,18,19], advanced keratoconus, fungal inflammation, and pterygium.

Recently, an MMP-9 point-of-care device (InflammaDry, Rapid Pathogen Screening Inc. Sarasota, FL, USA) demonstrated good agreement for confirming OSD. Patients with glaucoma often exhibit OSD, the incidence and severity of which are underestimated [20]. InflammaDry is a rapid test that detects elevated MMP-9 levels in tear fluid samples taken from the lower eyelid’s palpebral conjunctiva. It uses direct sampling microfiltration technology. Results are obtained in 10 min with high sensitivity (85%) and specificity (94%) [21]. A positive result indicates that the tear MMP-9 levels are >40 ng/mL [17].

The aim of the current study was to compare the potential ability of Latanoprost PF (0.005%) eye drops and Bimatoprost PF (0.03%) eye drops in promoting OSD in patients with primary open angle glaucoma (POAG). As mentioned above, the fact that BAK-preserved glaucoma eye drops promote OSD at higher rates than preservative-free eye drops is well described. To the best of our knowledge, this is the first attempt in the literature to compare two of the first-line and most prescribed treatments: preservative-free eye drops (Latanoprost PF and Bimatoprost PF).

## 2. Materials and Methods

### 2.1. Design

This prospective, non-blinded, single-center study was conducted at the 1st Department of Ophthalmology, National and Kapodistrian University of Athens, General Hospital of Athens “G. Gennimatas” in Athens, Greece. The study was conducted in accordance with the Declaration of Helsinki and was approved by the Ethics Committee of the General Hospital of Athens “G. Gennimatas” (Approval Code: 1831/23.01.2020).

### 2.2. Study Population

In total, 44 eyes from 44 participants were included in the study. We enrolled 24 participants in the control group and 20 participants who had been diagnosed with POAG, were enrolled in the POAG group. The POAG group was further divided into 2 sub-groups. Notably, 11 patients had been receiving only Latanoprost PF (0.005%) for at least 6 months, as glaucoma monotherapy and 9 patients only Bimatoprost PF (0.03%) as monotherapy for the same period of time. We chose to examine the right eye in all cases by default, since both eyes were treated equally in the POAG group and the control group eyes did not have any ocular pathology. The inclusion criteria for the control group were: (1) healthy eyes with no ocular pathology and (2) no instillation of any kind of eye drops, even artificial tears. The inclusion criteria for the POAG group were: (1) patients with diagnosed POAG and (2) patients who had been receiving Latanoprost PF or Bimatoprost PF as a monotherapy for at least 6 months. The exclusion criteria for all groups were as follows: (1) active inflammation or infection such as conjunctivitis, keratitis, or uveitis (2) Sjogren syndrome (3) a recent history of ocular surgery <6 months, (4) Meibomian gland dysfunction, (5) recent trauma, (6) contact lens use, (7) allergy, (8) diabetes mellitus (9) Instillation of artificial tear regularly.

### 2.3. Ocular Surface MMP-9 Levels, Schirmer’s Test and TBUT

The extracellular levels of MMP-9 were measured using the InflammaDry test (Rapid Pathogen Screening Inc. Sarasota, FL, USA) in all participants. The InfalmmaDry test was performed according to the manufacturer’s instructions. A positive result indicates that the MMP-9 levels in the tear fluid are >40 ng/mL. Additionally, we performed Schirmer’s test to all participants, with topical anaesthesia (0.5% proxymetacaine hydrochloride, Alcon Laboratories, Fort Worth, TX, USA). Finally, we estimated the TBUT in all examined eyes with the use of preservative-free fluorescein drops.

### 2.4. Statistical Analysis

The normal distribution of demographic and clinical information was assessed by plots (histogram and probability graphs) and corresponding statistical tests (Kolmogorov–Smirnov/Shapiro–Wilk test). Normally distributed continuous values were summarized by mean and standard deviation (SD) and discrete data by number (N) and percentage (%). We created different diagrams to show correlations among studied variables. Statistical significance was set at *p* < 0.05. Analysis was conducted in the Stata statistical software package version 13 (STATA Corp., College Station, TX, USA).

## 3. Results

### 3.1. Participants’ Demographics

In total, 44 participants were included in the study. 24 participants (54.55%) were enrolled in the control group and 20 patients (45.45%) in the POAG group. The POAG group was further subdivided into 2 sub-groups, 11 patients (25%) who had been receiving Latanoprost PF as monotherapy and 9 patients (20.45%) who had been receiving Bimatoprost PF as monotherapy. Overall, 26 (59.09%) of the participants were males and 18 (40.91%) were females. The mean age of the participants was 68.55 ± 10.66 years (range 43–87 years). The mean duration of treatment in months was 7.1 ± 0.83 for the Latanoprost PF group and 6.67 ± 0.71 for the Bimatoprost PF group. Participants’ demographics are summarized in Table 1.

### 3.2. Ocular MMP-9 Levels

We evaluated the MMP-9 levels in the tears of all participants using the InflammaDry kit test. The above testing revealed that 88.89% of glaucoma patients treated with Bimatoprost PF eye drops demonstrated clinically significant levels of MMP-9 (>40 ng/mL). On the contrary, only 27.27% of glaucoma patients treated with Latanoprost PF eye drops and 8.33% of the control group revealed elevated levels of MMP-9 in the tear film. This difference was statistically significant (*p* < 0.001). The above results are presented in Table 2 and Table 3.

### 3.3. Schirmer’s Test and TBUT

We performed Schirmer’s test and we evaluated the TBUT in all participants enrolled in the study. The Schirmer’s test values revealed a statistically significant difference between the control group and the Bimatoprost PF group, showing that controls had higher values than the glaucoma patients receiving Bimatoprost PF eye drops (*p*-value = 0.0076). Additionally, there was a statistically significant difference between the Latanoprost PF group and the Bimatoprost PF group, showing that Latanoprost PF users had higher Schirmer’s test values than the Bimatoprost PF users (*p*-value = 0.026). The difference between the controls and the Latanoprost PF users was not statistically significant.

**Figure 1:**
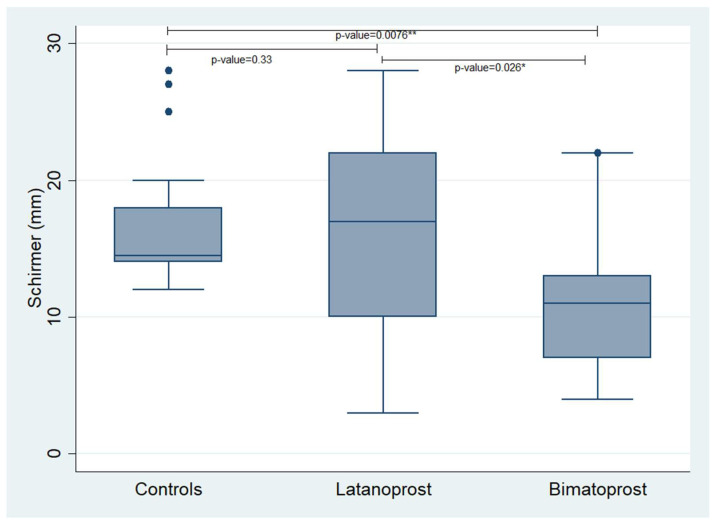
Box plot, presenting the values of Schirmer’s test in each studied group. *^,^** statistically significant values.

**Figure 2:**
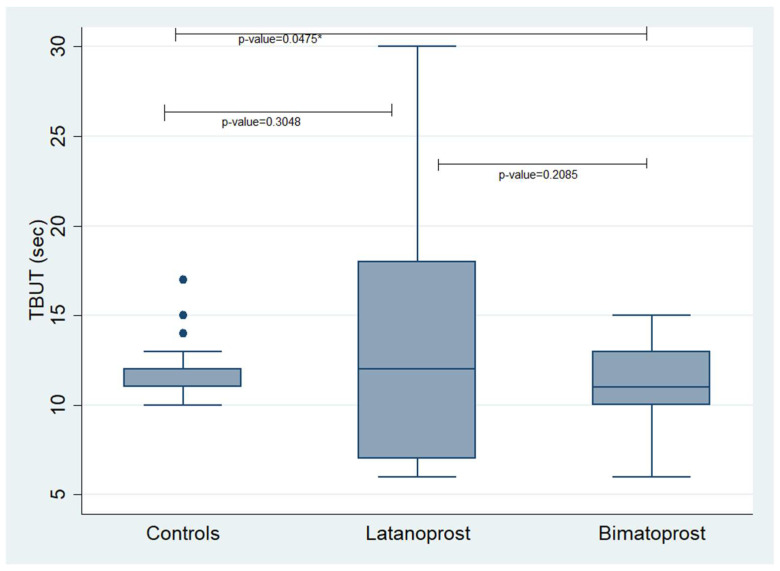
Box plot presenting the values of TBUT in each studied group. TBUT: Tear Break-Up Time. * statistically significant values.

Concerning the TBUT, we found a statistically significant difference between the controls and the Bimatoprost PF group showing that controls had better TBUT values compared to the Bimatoprost PF users (*p*-value = 0.0475). No other statistically significant correlations were revealed. The mean values of the examined variables in each studied group are presented in Table 3 and Table 4. Additionally, Figure 1 and Figure 2 demonstrate box plots and *p*-values of Schirmer’s and TBUT tests in each studied group, respectively.

## 4. Discussion

The major goals of glaucoma treatment are not only to control the IOP effectively but also to provide a good quality of life to the patient. The OSD symptoms are the second most common reason for switching medication, after low efficacy, which can lead to treatment failure and glaucoma progression [5]. Most antiglaucoma eye drops contain a preservative in addition to the active substance. Benzalkonium chloride (BAK) is the most frequently used preservative and studies have shown that BAK can affect the tear film and ocular surface by inducing squamous metaplasia of the conjunctival epithelium and by inducing proinflammatory cytokines along with a decrease in the number of goblet cells. The most frequently reported symptoms of ocular surface disease include burning and watery eyes, redness and blurred vision [22,23,24].

In order to avoid BAK-induced symptoms, we can administer preservative-free antiglaucoma eye drops. Previous studies have demonstrated that switching from a BAK-containing formulation to a preservative-free topical medication led to Schirmer’s test and TBUT improvement [25,26]. Subsequently, the management of the coexisting OSD in glaucoma patients is really important in order to maintain a good quality of life. Batra et al. have demonstrated that controlling OSD, resulted not only in the improvement of the OSD but also in better IOP control [27]. Non-adherence to glaucoma eye drops is a significant barrier to the successful treatment of glaucoma.

Prostaglandin analogues are the first-line treatment in patients with glaucoma and they are available in preserved as well as in preservative-free formulations with the same efficacy and better tolerability for PF solution [28]. Our study is the first to our knowledge that compares two of the most frequently prescribed preservative-free prostaglandin analogues (Latanoprost and Bimatoprost). The purpose of our study was to examine whether or not the above PF prostaglandin analogues induce OSD in patients with POAG. For that purpose, we evaluated the overexpression of MMP-9 with the InflammaDry test in all POAG patients and the control group, as well as Schirmer’s test and TBUT test. MMP-9 possesses a central role in the ocular inflammation procedure. MMP-9 activates the precursor IL-1β and latent TGF-Β1 into their active forms [29,30]. We noticed higher rates of MMP-9 overexpression in POAG patients receiving Bimatoprost PF medication (88.89%). On the contrary, 27.27% of the glaucoma patients receiving Latanoprost PF showed MMP-9 overexpression and only 8.33% of the control group revealed MMP-9 overexpression. The difference was statistically significant (*p* < 0.001). Concerning the Schirmer’s test and the TBUT test, the values in the Bimatoprost PF group were lower compared to the Latanoprost PF and the control group and the difference was statistically significant. Lower Schirmer’s test values represent reduced tear production and lower TBUT values are indicative of an impaired quality of the tear film.

Our findings are in accordance with similar studies. A previous meta-analysis that compared the preserved solutions of prostaglandin analogues, has shown that Latanoprost is associated with a lower incidence of conjunctival hyperemia when compared with Bimatoprost and Travoprost in the treatment of patients with glaucoma or ocular hypertension [31]. Zaleska-Zmijewska et al. reported that clinically significant MPP-9 levels (>40 ng/mL) were detected in the tear film from 46.7% of patients treated with preserved (BAK-containing) Latanoprost. In contrast, only 16.7% of patients treated with Tafluprost PF medication and untreated individuals demonstrated similar MMP-9 levels [4]. The above study did not compare the prostaglandin analogues that we compared but we can focus on the impact of BAK inducing the overexpression of MMP-9. In their study, 46.7% of patients receiving preserved Latanoprost revealed overexpression of MMP-9, whilst in our study only 27.27% of the patients did. Additionally, Kim et al. compared 67 patients with POAG, receiving topical preserved medication, with 47 healthy control subjects. MMP-9 overexpression was observed in 71.6% of the POAG group, whereas only 31.9% of the control group showed MMP-9 overexpression. Additionally, the POAG group was further subdivided according to the number of glaucoma medications received, 1, 2 or 3 bottles. The MMP-9 positivity to those subgroups was 25.0%, 40.9% and 61.9%, respectively. That finding suggests that the more preserved topical medications a patient receives, the higher the possibility of ocular inflammation occurring [32].

A possible explanation of our findings could be the different biochemistry of Bimatoprost compared to the Latanoprost molecule. Previous studies have categorized Bimatoprost in the group of prostamides [33,34]. On the contrary, other studies have shown that the Bimatoprost free acid activates the human F2a receptor similar to Latanoprost [35] and that Bimatoprost amide is hydrolyzed to the free acid by human cornea [36]. The above data prove that Bimatoprost is a prostaglandin analogue as Latanoprost, but with poor corneal penetration. This would explain the increased concentration of Bimatorpost (0.03%) compared with Latanoprost (0.005%).

The main limitation of our study is the limited number of cases examined. Enrichment of the study with more patients would be beneficial. As mentioned above, the preservative BAK is a well-known inducing factor of OSD, and our study was the first attempt to examine the potentially inducing OSD capability of prostaglandin analogues. In everyday clinical practice, we avoid prescribing prostaglandin analogues in neovascular glaucoma [37] or uveitic glaucoma [38], situations that implicate intraocular inflammation.

## 5. Conclusions

The current study is the first in literature that compares two of the most frequently prescribed and first-line treatments, preservative-free prostaglandin analogues (Latanoprost PF vs. Bimatoprost PF). We show that Latanoprost PF (0.005%) treats better the ocular surface than Bimatoprost PF (0.03%), as it does not induce the overexpression of the MMP-9 inflammatory molecule. Concerning Schirmer’s test, the Bimatoprost PF group revealed statistically significantly lower values compared to the control and the Latanoprost PF group, which indicates reduced tear production in the Bimatoprost PF group. Additionally, the TBUT values were statistically significantly lower in the Bimatoprost PF group compared to the control group, indicating an impaired tear film quality in the Bimatoprost PF group.

## Figures and Tables

**Table 1:** Study population demographics.

Variables	Controls (*n* = 24)	Latanoprost PF (*n* = 11)	Bimatoprost PF (*n* = 9)
Age ± SD	68.38 ± 12.15	69.9 ± 6.8	69 ± 10.1
Male/Female	14/10	7/4	5/4
Treatment Duration (months)	-	7.1 ± 0.83	6.67 ± 0.71

**Table 2:** Ocular MMP-9 levels in each studied group.

MMP-9 (<40 ng/mL)	Control (*n* = 24)	Latanoprost PF (*n* = 11)	Bimatoprost PF (*n* = 9)
Negative	22 (91.67%)	8 (72.73%)	1 (11.11%)
Positive	2 (8.33%)	3 (27.27%)	8 (88.89%)

Level of significance: *p*-value < 0.001.

**Table 3:** Baseline data of InflammaDry test, Schirmer’s test and Tear Break-Up Time Test in each studied group.

Patients	Group	InflammaDry	Schirmer’s Test (mm)	TBUT (s)
1	Control	Negative	14	10
2	Control	Negative	12	15
3	Control	Negative	12	14
4	Control	Negative	12	12
5	Control	Negative	15	13
6	Control	Negative	20	17
7	Control	Negative	18	11
8	Control	Positive	18	10
9	Control	Negative	15	11
10	Control	Negative	20	12
11	Control	Negative	18	12
12	Control	Negative	12	12
13	Control	Negative	14	12
14	Control	Negative	15	12
15	Control	Negative	14	12
16	Control	Negative	14	11
17	Control	Positive	14	11
18	Control	Negative	28	11
19	Control	Negative	27	11
20	Control	Negative	14	14
21	Control	Negative	13	12
22	Control	Negative	25	12
23	Control	Negative	14	12
24	Control	Negative	17	12
25	Latanoprost PF	Negative	22	9
26	Latanoprost PF	Positive	28	14
27	Latanoprost PF	Positive	9	6
28	Latanoprost PF	Negative	25	7
29	Latanoprost PF	Negative	3	12
30	Latanoprost PF	Negative	11	30
31	Latanoprost PF	Negative	10	18
32	Latanoprost PF	Negative	17	12
33	Latanoprost PF	Negative	12	9
34	Latanoprost PF	Positive	17	6
35	Latanoprost PF	Negative	17	19
36	Bimatoprost PF	Positive	4	7
37	Bimatoprost PF	Positive	13	13
38	Bimatoprost PF	Positive	22	15
39	Bimatoprost PF	Positive	7	10
40	Bimatoprost PF	Positive	12	13
41	Bimatoprost PF	Negative	20	11
42	Bimatoprost PF	Positive	5	10
43	Bimatoprost PF	Positive	8	6
44	Bimatoprost PF	Positive	11	12

**Table 4:** Mean values of Schirmer’s test and Tear Break-Up Time Test (TBUT) in each studied group.

	Control (*n* = 24)	Latanoprost PF (*n* = 11)	Bimatoprost PF (*n* = 9)
Schirmer’s Test (mm)	16.46 ± 4.6	15.55 ± 7.46	11.3 ± 6.28
TBUT (s)	12.12 ± 1.6	12.9 ± 7.2	10.78 ± 2.9

## Data Availability

Data available upon request.
